# Influence of Coronal Preflaring on the Accuracy of Electronic Working Length Determination: Systematic Review and Meta-Analysis

**DOI:** 10.3390/jcm10132760

**Published:** 2021-06-23

**Authors:** María León-López, Daniel Cabanillas-Balsera, Victoria Areal-Quecuty, Jenifer Martín-González, María C. Jiménez-Sánchez, Juan J. Saúco-Márquez, Benito Sánchez-Domínguez, Juan J. Segura-Egea

**Affiliations:** Endodontic Section, Department of Stomatology, University of Sevilla, C/Avicena s/n, 41009 Sevilla, Spain; maria.leon.lopez.98@gmail.com (M.L.-L.); victoria.areal@cabimer.es (V.A.-Q.); jmartin30@us.es (J.M.-G.); jimenezsanchez6@gmail.com (M.C.J.-S.); jjsauco@us.es (J.J.S.-M.); beni2506@yahoo.es (B.S.-D.)

**Keywords:** cervical preflaring, coronal preflaring, electronic apex locator, preflaring, root canal treatment, working length

## Abstract

Aim. To conduct a systematic review and meta-analysis according to the following PICO question: in extracted human permanent teeth, does preflaring, compared with unflared canals, influence the accuracy of WL determination with EAL? Material and Methods. A systematic review was conducted according to the PRISMA checklist, using the following databases: PubMed, Science Direct, Scopus, and Web of Science. Studies related to WL determination using EAL both in preflared and unflared root canals of extracted human teeth were included. The outcome of interest was the accuracy of the electronic WL determination. A quality assessment of the included studies was performed, determining the risk of bias. The meta-analyses were calculated with the 5.4 RevMan software using the inverse variance method with random effects. PROSPERO registration: CRD42021243412. Results. Ten experimental studies fulfilled the inclusion criteria, and most of them found that preflaring increases the accuracy of the EALs in WL determination. The calculated OR was 1.98 (95% CI = 1.65–2.37; *p* < 0.00001; I^2^ = 10%), indicating that the determination of WL by EALs is almost twice as accurate in preflared canals. The accuracy of Root ZX in WL determination increases more than three times (OR = 3.25; *p* < 0.00001). Preflaring with Protaper files significantly increases the accuracy of EALs (OR = 1.76; *p* < 0.00001). The total risk of bias of the included studies was low. No obvious publication bias was observed. Conclusions. The results indicate a significant increase in the accuracy of WL determination with EAL after preflaring, doubling the percentage of exact measurements. Preflaring should be recommended as an important step during mechanical enlargement of the root canal, not only because it improves the access of the files to the canal, but also because it allows one to obtain more accurate electronic determinations of WL.

## 1. Introduction

Initial passive exploration and scouting of the root canal using small stainless-steel K-files sizes, 06 to 10, in a watch-winding motion enables appreciation of the canal’s morphology and the presence of patency and potential resistance to file penetration [1]. Therefore, exploration and scouting should be the first step in root canal shaping. After initial exploration and scouting, a smooth radicular tunnel should be prepared from the canal orifice to the physiologic terminus (apical constriction), with this tunnel termed as the glide path [2]. However, to ensure that engine-driven files are used safely [3], negotiation and the glide path may be insufficient. A third operative step, known as preflaring, in both coronal and apical regions, is also necessary [4]. Apical preflaring has been defined as a pre-enlargement of the root canal up to its terminus using hand files to a size at least equal to the first engine-driven shaping fileinstrument that will be used [3]. However, to reduce the risk of rotary file separation when engaging in the root canal, it is also necessary to pre-enlarge the coronal third of the canal [5]. This operating step is called coronal preflaring [6]. Coronal preflaring reduces the contact between the file and the dentin walls, minimizing the torsional stress on the file. Moreover, coronal preflaring diminishes the initial coronal curvature, facilitating the access to the middle and apical thirds of the canal [4], and allows, from the start of the treatment, better penetration of the irrigant solution towards the apical third [6].

On the other hand, exact determination of the working length (WL) is a paramount factor both for the correct instrumentation and for obturation of the canal, and for the long-term success of root canal treatment [7]. The use of electronic apex locators (EALs) as an aid to determine the WL may perform better than radiography alone [8]. The WL determined by EAL decreases as a result of canal preparation [9]. Some studies have shown that one of the operative steps affecting WL is coronal preflaring [4,9,10,11]. However, De Moor et al. (1999) found that coronal preflaring did not ensure better or more precise WL readings [10]. On the contrary, Dean Davis et al. (2002) reported that, when initial WL was determined after coronal preflaring, few changes in the final WL were observed [9]. In summary, it is not clear to what extent preflaring influences the accuracy of final working length determination with electronic apex locators.

The aim of this study was to conduct a systematic review and meta-analysis according to the following PICO question: in extracted human permanent teeth (P), does coronal preflaring (I), compared with unflared canals (C), influence the accuracy of final WL determination with EAL (O)?

## 2. Materials and Methods

This systematic review is reported using the PRISMA guidelines [12] and the PICO framework. The review was registered in PROSPERO (CRD42021243412).

### 2.1. Literature Search Strategy

The search process was performed independently by four examiners (M.L.-L., D.C.-B., J.J.S.-M., and J.J.S.-E.). The electronic databases PubMed, Scopus, Dialnet, and Scielo were searched for articles published until 28 February 2021, without language, year restrictions or limits. Most cited descriptors in the previous publication on this theme were used in the electronic search strategy, using combining Medical Subject Heading (MeSH) terms and text word (tw). For each database, the following terms combinations were searched: (preflaring OR pericervical OR cervical enlargement) AND (root canal OR instrumentation OR cleaning and shaping OR endodontic treatment OR root-filled teeth OR root-filling OR obturation) AND (dentin OR dentine OR working length OR apical constriction). A complementary screening of the references of the selected studies was performed to find any additional study that did not appear in the primary database search.

### 2.2. Eligibility Criteria

Studies that evaluated the accuracy of WL determination using EAL both in preflared and unflared root canals of extracted human teeth were included. The eligibility criteria were based on the PICOS strategy [13] (PRISMA-P 2016), as follows:

- Population (P): extracted human permanent teeth;
- Intervention (I): coronal preflaring of root canals;
- Comparison (C): unflared root-canals;
- Outcome (O): accuracy of WL determination using EAL;
- Study design (S): laboratory.

The following were excluded: reviews, letters, opinion articles, conference abstracts, studies performed in animals, studies performed in humans, studies that included artificial teeth and studies in which it was not possible to calculate and compare the accuracy of WL determination with EAL in preflared and unflared canals.

### 2.3. Study Selection

Four authors (M.L.-L., D.C.-B., J.J.S.-M., and J.J.S.-E.) independently selected the retrieved studies by examining the titles and abstracts. When the title and abstract did not allow to judge the study, the full text was accessed. A second stage consisted of reading the full texts and judging the potential studies to be included based on the eligibility criteria through the PICOS strategy. Disagreements on study inclusion were solved by consensus with a fifth author (J.M.-G.). Duplicated studies in the database search were considered only once.

### 2.4. Data Collection/Extraction Process

Four authors (M.L.-L., D.C.-B., J.J.S.-M., and J.J.S.-E.) collected the data independently from the included studies. A fifth author (J.M.-G.) solved disagreements. Information regarding publication (author and publication year), study type, extracted teeth type, files used in coronal preflaring, the type of EAL used in the determination of WL, the reference for WL accuracy, percentages of accuracy in preflared and unflared canals, and the main result was extracted.

### 2.5. Quality Assessment and Risk of Bias of Individual Studies

Each selected study was evaluated for inner methodological risk of bias independently by four authors (M.L.-L., D.C.-B., B.S.-D., and J.J.S.-E.). Taking into account the fact that all included studies were “ex vivo” laboratory studies, a quality assessment was adopted following SYRCLE [14] with adaptations used in previous systematic reviews [15,16]. The following parameters were considered: (i) sample size calculation, (ii) samples with similar dimensions, (iii) control group, (iv) standardization of coronal preflaring, (v) standardization of EAL accuracy assessment of WL determination, and (vi) statistical analysis. The blinding of the operator was not taken into account, since the EAL type, file type and canals (unflared and preflared) are very different and allow the operator to identify the performed treatment. The parameters reported in original studies were scored dichotomously as (+) if present and (−) if missing. During the quality assessment, disagreements between authors were resolved through discussion with a fifth author (M.C.J.-S.). To assess the individual risk of bias, the articles were classified as having a low risk of bias if five or six items were reported, a moderate risk of bias if three or four items were reported, and a high risk of bias if only one or two parameters were reported. To assess the total risk of bias, the percentage of studies reporting each parameter was calculated, as well as the total percentage of the analyzed parameters that were reported in all the studies.

### 2.6. Outcome of Interest

The outcome of interest was the accuracy of the electronic working length determination in both unflared and preflared canals. The outcome was dichotomized according to whether the coronal preflaring significantly influenced the accuracy of WL determination, assessed electronically, or not. The odds ratio was calculated as a relative effect measure.

### 2.7. Data Synthesis and Statistical Analysis

The primary outcome measure was the accuracy of WL determination using EAL. The odds ratio (OR), with its 95% confidence interval (CI), was calculated in every selected study to measure the effect of preflaring on WL determination using EAL. The meta-analysis was carried out on studies that provided data, or where it was possible to calculate data, on the percentages of accuracy in WL determination with EAL in unflared and preflared canals. In some of the included studies, accuracy was calculated as the percentage of exact matches with the apical constriction, while in others, a margin of ±0.5 mm was allowed. Studies that compared the accuracy of electronic WL determination but only reported data on means and standard deviations, not allowing accuracy calculation, were excluded.

Two subgroup analyses were performed, one including the studies using Protaper files (Dentsply Maillefer, Ballaigues, Switzerland) for preflaring, and another grouping all studies using the Root ZX (J Morita Corp, Tokyo, Japan) apex locator for WL determination.

The meta-analyses were calculated with the 5.4 RevMan software (Review Manager Web. The Cochrane Collaboration, 2019. Available at revman.cochrane.org, accessed on 24 May 2021). The studies did not conduct the assays using the same methodology—on the contrary, different types of preflaring files and EALs were used. The inverse variance method with random effects was thus performed to determine the pooled OR and its 95% CI. Meta-analyses were represented with a forest plot [17].

To estimate the variance and heterogeneity amongst trials, the Tau^2^ and the Higgins I^2^ tests were employed, considering a slight heterogeneity if the value was between 25 and 50%, moderate between 50% and 75%, and high if >75% [18]. The existence of statistical significance was assessed using the Z test (*p*-value < 0.05). A funnel plot was plotted to illustrate the possible existence of publication bias [19].

## 3. Results

### 3.1. Selection of the Studies

**Figure 1:**
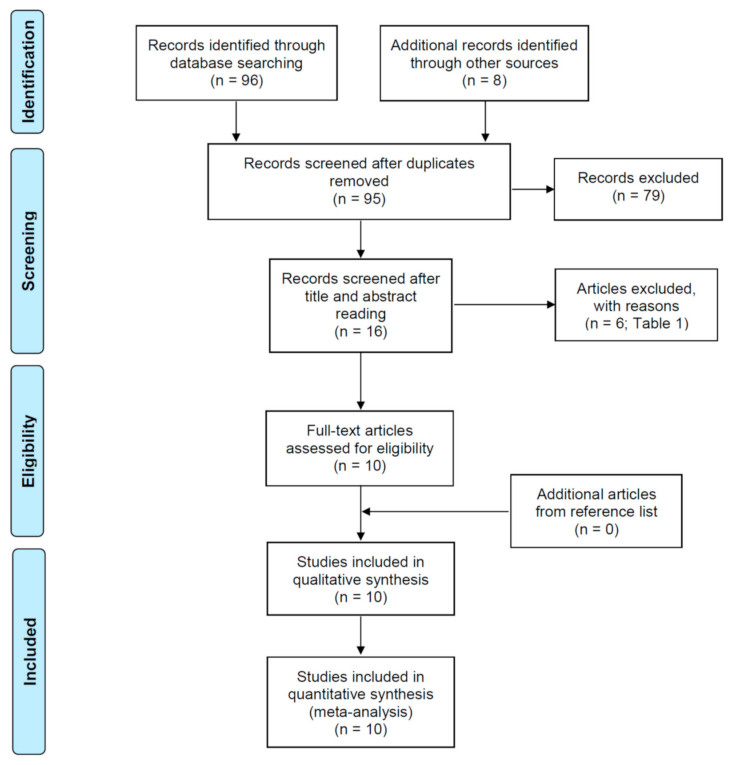
Flow diagram of the search strtegy of the systematic review and meta-analysis following the Preferred Reporting Items for Systematic Reviews and Metaanalyses (PRISMA) guidelines.

The flow diagram of the search strategy is shown in Figure 1. The initial search resulted in 96 published studies from different databases, together with 8 additional studies identified through other sources. Nine studies were excluded as they were duplicates. Then, from 95 eligible papers, the analysis of titles and abstracts resulted in the inclusion of 16 studies of interest. The reason for the rejection of 79 articles was that they did not match the inclusion criteria, as they did not relate coronal preflaring with the EAL accuracy in the determination of the working length. After comprehensive reading, 10 full-text articles were selected for the systematic review and meta-analysis [20,21,22,23,24,25,26,27,28,29], and 6 articles were excluded for different reasons [10,30,31,32,33,34] (Table 1).

### 3.2. Characteristics of the Included Studies

The data collected from the ten included studies are summarized in Table 2. All the included studies compared the accuracy of different EALs in WL determination in unflared and preflared teeth. Seven studies used Root ZX [20,21,23,25,26,28,29]. The most used instruments for preflaring were Protaper SX and S1 files [21,23,25,27,28], followed by LA Axxess burs (SybronEndo, Glendora, CA, USA) [22,25]. All the included studies used similar methodologies when preparing coronal preflared and unflared teeth and during WL determination with EALs. However, some studies considered the measurements of WL that exactly coincided with the apical constriction as accurate [20,21,23,24,26], and others took the measurements ± 0.5 mm from apical constriction as accurate [22,25,27,28,29]. The included studies used different types of teeth: incisors and canines [21,22,25,27,28], premolars [28], and molars [20,23,24,29].

Most of the studies found that preflaring increased the accuracy of the EALs, although the differences between the accuracy in unflared and preflared canals were not always significant.

### 3.3. Outcomes of the Primary Meta-Analysis and Publication Bias

To carry out the meta-analysis, the results of the included studies were divided into sections according to the number of EALs used and the type of files with which the preflaring was carried out. In total, 34 results were included in the meta-analysis, including 2890 electronic determinations of working length, half in unflared canals and the other half in preflared canals.

**Figure 2:**
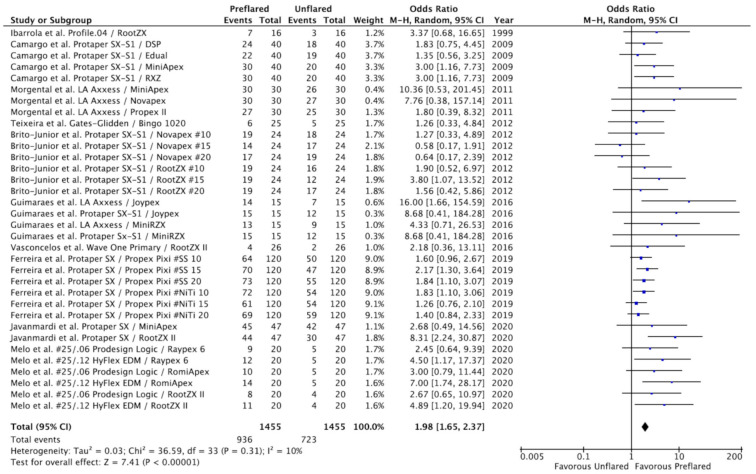
Forest plot of ORs and 95% confidence limits (CL) for the comparison of unflared and preflared canals regarding the accuracy of electronic apex locators (EALs) in working length (WL) determination. The overall estimate is based on the data from the ten included studies. Black squares represent the point estimate of the odds ratio and have areas proportional to study size. Lines represent 95% confidence intervals. The diamond shows the summary statistic for the 34 results from the ten included studies. The solid line indicates an odds ratio of 1.0. OR: odds ratio.

The estimated variance among all results was examined by the Tau^2^ test, with the result being not significant (Tau^2^ = 0.03; chi^2^ = 36.5; df = 33; *p* = 0.31). The heterogeneity test value (I^2^ = 10%) was low; however the weights were calculated using the random effects model, considering that there was variation among the included studies and allowing the study outcomes to vary in a normal distribution. Overall OR was 1.98 (95% CI = 1.65–2.37; *p* < 0.00001), indicating that the determination of WL by EALs was significantly more accurate in preflared canals compared to unflared canals. The ORs for the 34 results of the ten included studies and the pooled OR from the meta-analysis are shown in a forest plot (Figure 2).

**Figure 3:**
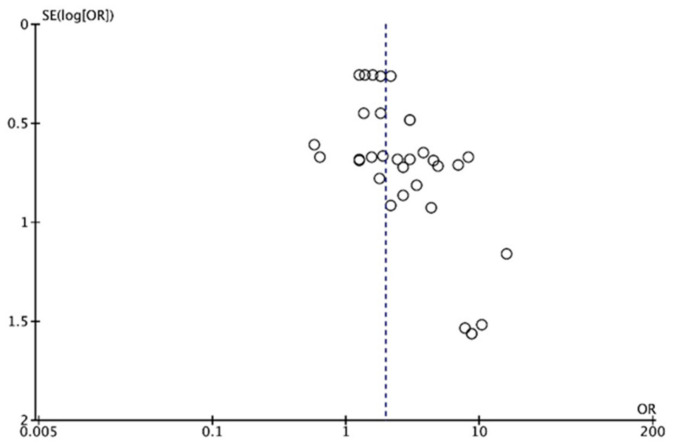
Funnel plot. Each dot indicates one of the 34 results from the ten included studies. The y axis represents the standard error (SE) of the OR, and the x-axis represents the OR calculated in the meta-analysis.

Figure 3 shows a funnel plot of the eligible studies. The eligible articles are distributed evenly around the vertical line that indicates the summary estimate, and studies with higher power and lower standard error are plotted towards the top and low powered studies are placed near the bottom. No obvious publication bias was observed.

### 3.4. Additional Analysis

**Figure 4:**
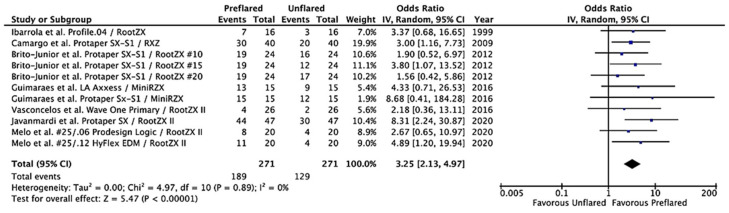
Forest plot of ORs and 95% confidence limits (CL) for the comparison of unflared and preflared canals regarding the accuracy of Root ZX in working length (WL) determination. The overall estimate is based on the data from the seven included studies. Black squares represent the point estimate of the odds ratio and have areas proportional to study size. Lines represent 95% confidence intervals. The diamond shows the summary statistic for the 11 results from the seven included studies. The solid line indicates an odds ratio of 1.0. OR: odds ratio.

Considering that Root ZX was the most used EAL in the included studies, a meta-analysis using the inverse variance with the random effects model was carried out including only the results of the Root ZX [20,21,23,25,26,28,29]. The result of the Tau^2^ test was not significant (*p* = 0.89), with 0% heterogeneity. Calculated overall OR was 3.25 (95% CI = 2.13–4.97; *p* < 0.00001), indicating that the accuracy of Root ZX for WL determination increases significantly, more than three times, in preflared canals (Figure 4).

**Figure 5:**
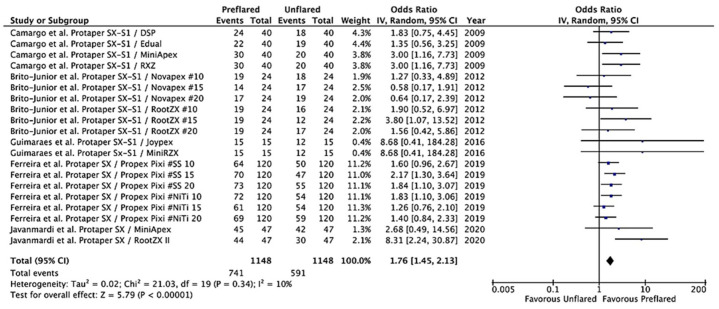
Forest plot of ORs and 95% confidence limits (CL) for the comparison of unflared and preflared canals using Protaper SX and S1 files regarding the accuracy of EALs in working length (WL) determination. The overall estimate is based on the data from the seven included studies. Black squares represent the point estimate of the odds ratio and have areas proportional to study size. Lines represent 95% confidence intervals. The diamond shows the summary statistic for the 20 results from the five included studies. The solid line indicates an odds ratio of 1.0. OR: odds ratio.

Since Protaper files, SX and S1, were the most used for coronal preflaring in the included studies, a new meta-analysis, using the same model, was carried out including only the results of the five studies that performed preflaring with Protaper files [21,23,25,27,28]. The Tau^2^ test was not significant (*p* = 0.34), and heterogeneity was 10%. The calculated overall OR was 1.76 (95% CI = 1.45–2.13; *p* < 0.00001), indicating that coronal preflaring of canals using Protaper Sx and S1 significantly increases the accuracy of EALs in the termination of WL (Figure 5).

### 3.5. Quality Assessment and Risk of Bias

**Figure 6:**
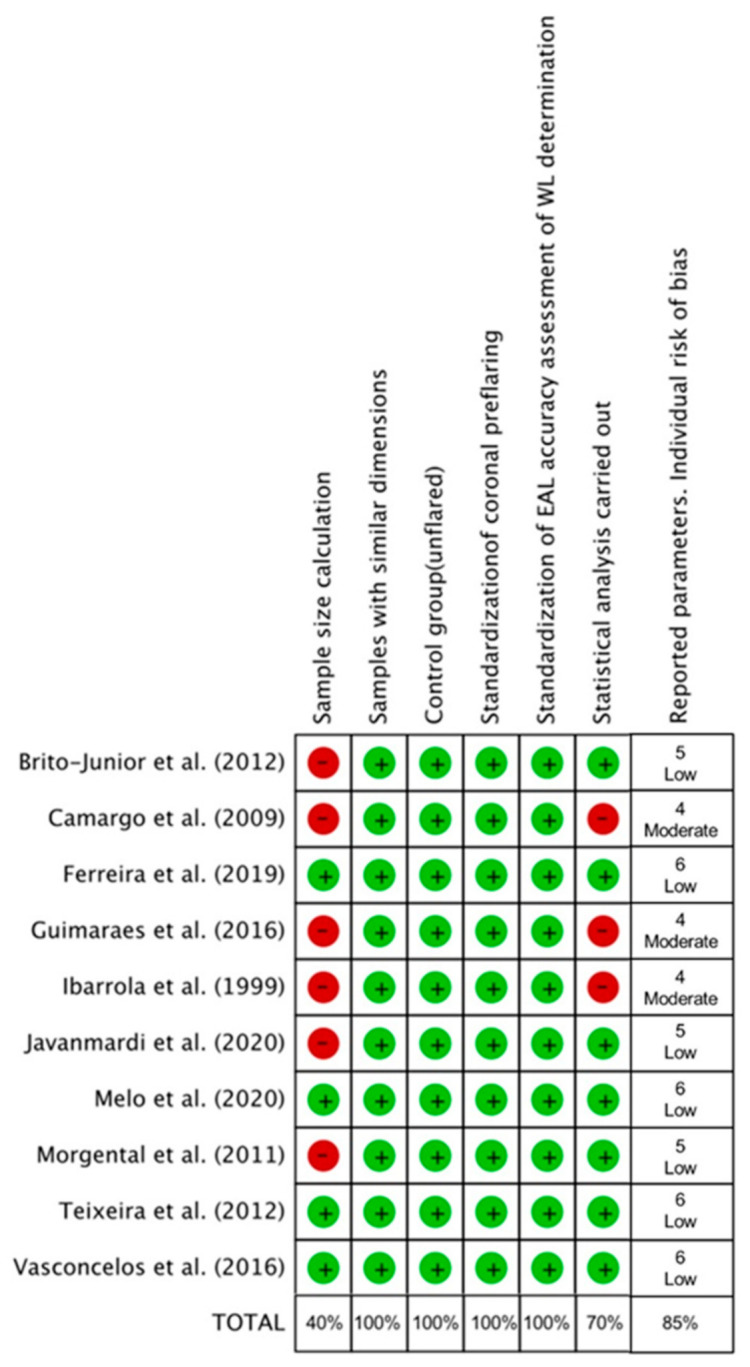
Quality assessment of included studies according to SYRCLE’s risk of bias tool [14], a RoB tool for animal intervention studies based on the Cochrane Collaboration RoB Tool [35].

The methodological quality and the risk of bias of each study were assessed (Figure 6). According to the parameters considered in the analysis, three of the included studies presented moderate risk of bias [20,21,25], three others reported five of the six analyzed parameters [22,23,28], meaning they were classified as low risk of bias, and the remaining four studies [24,26,27,29] reported all of the analyzed parameters, and therefore they were also considered as low risk of bias. In the present analysis, four of the six parameters assessed (samples with similar dimensions, control group (unflared), standardization of coronal preflaring, and standardization of EAL accuracy assessment of WL determination) were reported in all the included studies. The total percentage of parameters reported in the 10 included studies was 85%, indicating a low total risk of bias.

## 4. Discussion

The present study aimed to analyze the influence of coronal preflaring on the accuracy of electronic WL determination. The results of the systematic review and meta-analysis carried out, including the available evidence comparing the accuracy of EALs in unflared and preflared root canals, conclude that preflaring increases the accuracy of EALs, doubling the percentage of exact values. To our knowledge, this is the first systematic review analyzing the influence of coronal preflaring in the accuracy of WL determination with EALs, a topic that has not been investigated so far by meta-analysis. Thus, the result of the present study fills this knowledge gap and should be considered very relevant in the root canal treatment protocol.

Considering that Root ZX was the most used EAL in the studies included in the meta-analysis [20,21,23,25,26,28,29], the inverse of the variance method with random effects was also used to calculate the overall OR for the effect of the coronal preflaring in the accuracy of Root ZX. The result (OR = 3.25; 95% CI = 2.13–4.97; *p* < 0.00001) indicates that the accuracy of Root ZX for WL determination increases more than three times after preflaring.

### 4.1. Implications for Practice and Research

The results of this meta-analysis have very important implications for the daily dental clinic. Certainly, coronal preflaring is well known as an important operative maneuver to achieve the so-called straight-line approach to the canal orifice and its first curvature [4]. This minimizes the errors during subsequent treatment procedures. However, after the results of the present study, preflaring should also be considered an important step to accurately determine the WL using EALs. Therefore, the present results should be taken into account during mechanical enlargement of the root canal, knowing that coronal preflaring will not only improve the access of the files to the canal, but it will also help to achieve a more accurate electronic WL determination. On the other hand, to perform a safe crown-down instrumentation of curved canals, without overflaring of the pericervical dentin, Elkholy and Ha (2021) [35] proposed a novel instrument technique [35], recommending three apical strokes after the initial engagement before withdrawal to minimize instrumentation time.

### 4.2. Quality Assessment

After the literature search, ten studies that met the inclusion criteria and provided data about the accuracy of EALs in unflared and preflared canals were included [20,21,22,23,24,25,26,27,28,29], including a very high number of measurements: 1445 electronic determinations of WL in unflared canals and another 1445 in preflared canals. All included articles reported “ex vivo” studies performed in the laboratory using human extracted teeth. Therefore, to assess their methodological quality, the SYRCLE’s risk of bias tool [14] was taken as a reference, with adaptations [16]. Systematic Review Centre for Laboratory Animal Experimentation (SYRCLE) is a RoB tool for animal intervention studies based on the Cochrane Collaboration RoB Tool [36]. SYRCLE contains entries related to selection bias, performance bias, detection bias, attrition bias, reporting bias and other biases. The adaptation for the present systematic review considered six parameters: (i) sample size calculation, (ii) samples with similar dimensions, (iii) control group, (iv) standardization of coronal preflaring, (v) standardization of EAL accuracy assessment of WL determination, and (vi) statistical analysis. Moreover, of the six parameters used to assess the risk of bias, the 10 included studies reported adequately 85%, indicating a low total risk of bias. No obvious publication bias was observed. Therefore, there is a lot of confidence that the true effect is similar to the estimated effect, i.e., that coronal preflaring increases the accuracy of EALs.

Regarding the meta-analysis, although the heterogeneity of the studies was very low (I^2^ = 10%), taking into account the fact that the included studies used different types of preflaring files and EALs, the inverse variance method with random effects was performed. The overall OR value obtained was 1.98 (95% CI = 1.65–2.37; *p* < 0.00001), indicating that the determination of WL by EALs was significantly more accurate in preflared canals compared to unflared canals.

### 4.3. Strength and Limitations

Among the strengths of this systematic review and meta-analysis, one is the use of the random-effects model. This model explicitly accounts for the heterogeneity of studies through a statistical parameter representing the inter-study variation [37]. The random-effects model assumes that the true effect size may or may not vary from study to study, i.e., there is a distribution of true effects. The overall effect is an estimate of that distribution’s mean. Therefore, the result of the present study indicates that preflaring actually improves the precision of the EALs to determine the WL. Furthermore, the high number of electronic WL measurements included in the meta-analysis (2890) and the overall low risk of bias can also be considered strengths of this systematic review and meta-analysis.

However, the present systematic review also has several limitations. One of these limitations refers to the fact that six of the included studies did not include a sample size calculation in their methodology [20,21,22,23,25,28], with high risk of bias in this issue. Another limitation of the present study is that the grey literature was not systematically searched, although all the references of the included articles were analyzed, including articles in Spanish and Portuguese. A key point in the topic analyzed in this systematic review is the performance of the coronal preflaring. The number and taper of the file used in preflaring may influence the enlargement of the coronal portion of the canal. The fact that the files used to perform the coronal enlargement are different in each study could also be considered a limitation. Among the files used for coronal preflaring, Protaper SX and S1, which have the smallest size and taper in their tips, although their progressive taper makes their coronal caliber large, were used in five studies [21,23,25,27,28]. The calculated overall OR for these five studies was 1.76 (*p* < 0.00001), a value lower than the overall OR calculated for the 10 studies. This is consistent with the fact that the other five studies included in the review used larger sizes and greater taper (20/0.06) [22,25], or Prodesign Logic (25/0.06 (Prodesign Logic; Bassi Endo Product, Belo Horizonte, MG, Brazil)) and HyFlex EDM (25/0.12) (Coltene-Whaledent, Allstätten, Switzerland) [29]. Finally, another limitation of the present systematic review is that the included studies used different criteria to determine the accuracy of the electronic WL. Some studies considered the measurements of WL that exactly coincided with the apical constriction as accurate [20,21,23,24,26], and others took the measurements ± 0.5 mm from apical constriction as accurate [22,25,27,28,29]. However, in all studies, the accuracy of the measurement was determined in coronal preflared and unflared canals.

## 5. Conclusions

The results of the available studies indicate a significant increase in the accuracy of WL determination with EAL after coronal preflaring, doubling the percentage of exact measurements. Therefore, coronal preflaring should be recommended as an important step during the mechanical enlargement of the root canal, not only because it improves the access of the files to the canal, but also because it allows one to obtain more accurate electronic determinations of WL.

## Figures and Tables

**Table 1:** Excluded studies and the respective reasons for each exclusion.

Reasons for Exclusion	Authors and Year
Accuracy was not reported/cannot be determined	De Moor et al. 1999 [10] Tinaz et al. 2002 [30] Suryantoro et al. 2017 [33] Maniglia-Ferreira et al. 2017 [34]
EAL not used	Iqbal et al. 2013 [31] Kumar et al. 2013 [32]

**Table 2:** Summary of descriptive characteristics of included studies.

Authors Year	Extracted Teeth Used	Files Used in Preflaring	EAL Used for WL	Reference for WL Accuracy	Accuracy in Unflared Canals (%)	Accuracy in Preflared Canals (%)	Main Result
Ibarrola et al. 1999	16 lower molars; 32 mesial canals (Weine III)	Profile 04 sizes 9 to 6	Root-ZX	Exact apical constriction	RZX—18.8%	RZX—43.8%	Preflaring significantly increased the accuracy of the WL determination with EAL (*p* = 0.015).
Camargo et al. 2009	40 lower incisors (Vertucci I)	Protaper SX Protaper S1	Root-ZX Edual Mini Apex DSP	Exact apical constriction	RZX—50% EduaL—47.5% Mini Apex—50% Apex DSP—45%	RZX—75% Edual—55% Mini Apex—75% Apex DSP—60%	Preflaring significantly increased the precision to determine the real WL with Root ZX and Mini Apex (*p* > 0.05), but no significant difference was noted for Edual and Apex DSP (*p* > 0.05).
Morgental et al. 2011	30 lower incisors	LA Axxess 20/0.06	Novapex Mini Apex ProPex II	±0.5 mm from apical constriction	Novapex—90% Mini Apex—87% Propex II—83%	Novapex—100% Mini Apex—100% Propex II—90%	Preflaring increased the accuracy of Mini Apex and Propex II (*p* < 0.05), but no significant difference was noted for Novapex (*p* > 0.05).
Brito-Junior et al. 2012	24 upper molars	Protaper SX Protaper S1	Novapex	Exact apical constriction	#10–75% #15–70% #20–80%	#10–80% #15–60% #20–70%	Coronal preflaring did not increase accuracy in the electronic measurements (*p* > 0.05).
Brito-Junior et al. 2012	24 upper molars	Protaper SX Protaper S1	Root-ZX	Exact apical constriction	#10–65% #15–50% #20–70%	#10–80% #15–80% #20–80%	Coronal preflaring significantly increased accuracy in the electronic measurements (*p* < 0.05).
Teixeira et al. 2012	25 lower molars; 50 canals	Gates-Glidden 4-3-2	Bingo1020	Exact apical constriction	Bingo1020—21%	Bingo1020—25%	Preflaring with Gates Glidden drills were not able to significantly influence the accuracy of the apex locator in determining the exact working length (*p* > 0.05).
Guimaraes et al. 2016	15 lower incisors (Vertucci I)	Protaper SX Protaper S1	Joypex 5 RZX Mini	±0.5 mm from apical constriction	Joypex 5—80% RZX Mini—80%	Joypex 5—100% RZX Mini—100%	Both EALs presented a higher percentage of exact measurements after preflaring, but differences were not significant (*p* > 0.05).
Guimaraes et al. 2016	15 lower incisors (Vertucci I)	LA Axxess 20/0.06	Joypex 5 RZX Mini	±0.5 mm from apical constriction	Joypex—46.6% RZX Mini—60%	Joypex—93.3% RZX Mini—86.6%	Cervical preparation with LA-Axxes increased the accuracy of the EAL Joypex (p = 0.01), but not of the RZX Mini (*p* > 0.05).
Vasconcelos et al. 2016	26 lower molars (Vertucci IV); 52 canals	WaveOne Primary	Root ZX II	Exact apical constriction	RootZX II—7.7%	RootZX II—15.4%	The accuracy of Root ZX II presented no change considering the time interval when the electronic measurement was made. (*p* > 0.05)
Ferreira et al. 2019	40 upper anterior teeth (Vertucci I).	Protaper SX	Propex Pixi	±0.5 mm from apical constriction	SS files 10 mm—41.7% 15 mm—39.1% 20 mm—45.9%	SS files 10 mm—53.4% 15 mm—58.3% 20 mm—60.8%	Preflaring procedures increase the accuracy of Propex Pixi regardless of the size of the SS file (*p* < 0.05).
Ferreira et al. 2019	40 upper anterior teeth (Vertucci I).	Protaper SX	Propex Pixi	±0.5 mm from apical constriction	NiTi files 10 mm—45% 15 mm—45% 20 mm—49.2%	NiTi files 10 mm—60% 15 mm—50.8% 20 mm—57.5%	Preflaring procedures increase the accuracy of Propex Pixi regardless of the size of the NiTi file (*p* < 0.05).
Javanmardi et al. 2020	47 teeth (11 incisors, 10 canines and 26 premolars); 60 canals in total	Protaper SX	Root ZX II	±0.5 mm from apical constriction	RootZX—63.3%	Root ZX—93.3%	Preflaring increases the accuracy of RZX in the determination of WL (*p* < 0.001).
Javanmardi et al. 2020	47 teeth (11 incisors, 10 canines and 26 premolars); 60 canals	Protaper SX	Mini Apex Locator	±0.5 mm from apical constriction	Mini Apex—90%	Mini Apex—96.6%	Preflaring does not increase the accuracy of Mini Apex in the determination of WL (*p* = 0.293).
Melo et al. 2020	20 lower molars (Vertucci IV)	Prodesign Logic 25/0.06	Root ZX II Raypex 6 RomiApex A-15	±0.5 mm from apical constriction	Root ZX—20% Raypex 6—25% RomiApex—25%	Root ZX—40% Raypex 6—42.5% RomiApex—50%	An improvement in the accuracy of EALs after conventional coronal preflaring enlargement was observed (*p* < 0.05).
Melo et al. 2020	20 lower molars (Vertucci IV)	HyFlex EDM 25/0.12	Root ZX II Raypex 6 RomiApex	±0.5 mm from apical constriction	Root ZX II—20% Raypex 6—25% RomiApex—25%	Root ZX II—55% Raypex 6—57.5% RomiApex—70%	An improvement in the accuracy of EALs after conventional coronal preflaring enlargement was observed (*p* < 0.05)

Profile, Protaper and Wave One (Dentsply Maillefer, Ballaigues, Switzerland). LA Axxess (SybronEndo, Glendora, CA, USA). Prodesign (Prodesign Logic; Bassi Endo Product, Belo Horizonte, MG, Brazil). Hyflex EDM (Coltene-Whaledent, Allstätten, Switzerland). Root ZX (RZX) and Root ZX Mini (J Morita Corp, Tokyo, Japan). Propex II and Propex Pixi (Dentsply Maillefer, Ballaigues, Switzerland) Raypex 6 (VDW GmbH, Munich, Germany). RomiApex A-15 (Romidan Ltd., Kiryat Ono, Israel). Mini Apex Locator (SybronEndo, Anaheim, CA, USA). (Denjoy, Changsha, China). Bingo 1020 (Forum Engineering Technologies, RishonLezion, Israel). Edual (Sybron Dental, Sybron Dental, Anaheim, CA, USA). Apex DSP (Septodont, Saint-Maur des Fosse’s, Cedex, France. Novapex (Forum Technologies, Rishon Le-Zion, Israel).
